# Exploring the longitudinal expression dynamics of midguts in adult female *Amblyomma americanum* ticks

**DOI:** 10.1186/s12864-024-10905-y

**Published:** 2024-10-24

**Authors:** Stephen Lu, Lucas C. de Sousa-Paula, Jose M. C. Ribeiro, Lucas Tirloni

**Affiliations:** 1 Vector Biology Section, Laboratory of Malaria and Vector Research, National Institute of Allergy and Infectious Diseases, Bethesda, MD USA; 2 Tick-Pathogen Transmission Unit, Laboratory of Bacteriology, National Institute of Allergy and Infectious Diseases, Hamilton, MT USA

**Keywords:** Ticks, Blood meal digestion, Midgut, RNA-sequencing

## Abstract

**Background:**

Female ticks remain attached to their host for multiple days to complete a blood meal. This prolonged feeding period is accompanied by a significant increase in the tick’s size and body weight, paralleled by noteworthy changes to the tick midgut. While the midgut is recognized for its established role in blood storage and processing, its importance extends to playing a crucial role in the acquisition, survival, and proliferation of pathogens. Despite this, our overall understanding of tick midgut biology is limited.

**Results:**

Our transcriptome analysis identified 15,599 putative DNA coding sequences (CDS), which were classified into 26 functional groups. Dimensional and differential expression analyses revealed four primary transcriptional profiles corresponding to unfed, slow-feeding, transitory (from slow- to rapid-feeding), and rapid-feeding stages. Additionally, comparing the current dataset with previously deposited transcriptome from other tick species allowed the identification of commonly expressed transcripts across different feeding stages.

**Conclusion:**

Our findings provide a detailed temporal resolution of numerous metabolic pathways in the midgut of *A. americanum* adult females throughout the feeding process, highlighting the dynamic transcriptional regulation of the tick’s midgut as feeding progresses. Furthermore, we identified conserved transcripts across three different tick species that exhibit similar expression patterns. This knowledge not only enhances our understanding of the physiological processes within the tick midgut but also opens up potential avenues for developing control methods that target multiple tick species.

**Supplementary Information:**

The online version contains supplementary material available at 10.1186/s12864-024-10905-y.

## Introduction

*Amblyomma americanum*, commonly known as the lone star tick, is a three-host tick species prevalent in the eastern, southeastern and south-central regions of the United States [1, 2]. The increasing presence of *A. americanum* raises concerns for public health, given its role as a vector for various pathogens. Specifically, it has been implicated in the transmission of *Erlichia chafeensis* [3], the causative agent of human monocytic ehrlichiosis, *Francisella tularensis*, responsible for tularemia [4], and *Borrelia lonestari* [5], linked to the southern tick-associated rash illness. Recently, this tick species has been proposed as the primary vector for Heartland and Bourbon viruses [6]. Furthermore, bites of *A. americanum* ticks have been associated with the development of alpha-gal syndrome [7], highlighting its growing significance for public health.

The feeding cycle of an adult female tick unfolds in three distinct stages. The initial preparatory phase involves the tick attaching to its host’s skin and forming a lesion, setting the stage for the acquisition of its blood meal. The subsequent stage is commonly known as the slow-feeding phase, extending over multiple days. During this phase, the tick experiences a gradual increase in body size and mass as it consumes the host’s blood. The final stage, termed the rapid-feeding phase, occurs in the last 12 to 24 h of feeding and is characterized by the swift ingestion of host blood [8]. Throughout the feeding process, the tick midgut displays notable morphological changes [9], underscoring its high plasticity.

Beyond its established role in blood meal storage and processing [8, 10], the tick midgut serves as the primary entry point for pathogens, in which key interactions between pathogen and tick molecules are necessary to ensure the survival and proliferation of such pathogens [11, 12]. Consequently, different research groups have focused on comprehensively exploring the composition of the tick midgut [13–15] to gain a better understanding of the physiological process within. Furthermore, studies have demonstrated that targeting midgut proteins could be an effective strategy for tick control [16, 17].

It is important to acknowledge that, despite the recent efforts to comprehend tick midgut physiology, the majority of studies have predominantly focused on the early stages of feeding [18, 19]. Consequently, these investigations offered a limited perspective on the dynamic changes occurring within the tick midgut throughout the entire feeding process. To bridge this gap, we previously conducted a midgut transcriptome analyses of *Ixodes scapularis* and *Rhipicephalus microplus* adult female ticks [20, 21]. These studies highlighted the presence of specific transcriptional profiles associated with the different feeding stages, with hundreds of transcripts being modulated as feeding progresses. Furthermore, it provide insights into the genetic mechanisms involved in blood meal processing in ticks. Additionally, these studies served as a foundation for comparative studies among different tick species, which may help in identifying targets for the development of anti-tick control strategies.

Here, we present an in-depth analysis of the transcriptional alterations observed in the midgut of *A. americanum* adult females during feeding. Recognizing the significance of targeting proteins in the early feeding stages, we also compared findings from the *A. americanum* midgut transcriptome with those of *I. scapularis* [20] and *R. microplus* [21]. Orthology analyses were performed to identify genes that are shared in different feeding phases among these tick species. Altogether, this dataset not only enhances our understanding of the adaptations of the tick midgut to the blood meal but also contributes to the identification of potential novel targets for the development of anti-tick control methods.

## Materials and methods

### Ethics statement

Animal experiments were conducted in accordance with the guidelines of the National Institutes of Health on protocols approved by the Rocky Mountain Laboratories Animal Care and Use Committee (2020-065). The Rocky Mountain Veterinary Branch is accredited by the International Association for Assessment and Accreditation of Laboratory Animal Care (AAALAC).

### Tick rearing and midgut dissection

*Amblyomma americanum* ticks were purchased from the tick rearing facility at Oklahoma State University. Unfed ticks were maintained at 21 °C and 80–90% relative humidity before infestation. Adult ticks used for midgut extraction were restricted to feed onto the outer part of the ear of four naïve female New Zealand White rabbits with glued orthopedic stockinet. A total of 15 adult females and 15 males (30 ticks per ear, 60 ticks per animal) were placed into the tick containment apparatus and allowed to attach. To group ticks by a blood feeding index, partially fed ticks were collected from host during the feeding, selected based on their engorgement size, and sorted by their average weight: group unfed (UF, 4.7 ± 0.62 mg, 10 ticks per sample), group 1 (G1: 6.4 ± 0.60 mg, 5 ticks per sample), group 2 (G2: 16.4 ± 1.82 mg, 5 ticks per sample), group 3 (G3: 24.7 ± 3.24 mg, 5 ticks per sample), group 4 (G4: 67.2 ± 7.30 mg, 5 ticks per sample), group 5 (G5: 373.9 ± 34.48 mg, 3 ticks per sample), and group 6 (G6: 577.0 ± 88.50 mg, 3 ticks per sample). All biological groups (unfed, G1, G2, G3, G4, G5 and G6) were collected in triplicates, resulting in a total of 21 samples. After removal from the host, ticks were rinsed with 1% bleach, nuclease-free water, and 70% ethanol, followed by a final rinse with nuclease-free water. Ticks were dissected within two hours after removal from the host. Tick midguts (MGs) were dissected in a fresh ice-cold nuclease-free phosphate-buffered saline (PBS), pH 7.4 (Invitrogen). After dissection, MGs were gently washed in fresh nuclease-free PBS, pH 7.4, containing 4 U/mL of RNAse inhibitor (RNaseOUT, Thermo Fisher Scientific) and a protease inhibitor cocktail (Sigma Aldrich). After washing, dissected MGs were immediately stored in RNAlater (Invitrogen) until total RNA extraction.

### Library preparation, sequencing, and data analysis

Total RNA was isolated using the AllPrep DNA/RNA/Protein mini kit (QIAGEN) according to the manufacturer’s instructions. RNA integrity and quantification were assessed using a 4200 TapeStation system (Agilent Technologies). The directional Illumina libraries were constructed using the NEBNextUltraTM II RNA with polyA selection library prep kit. Sequencing was performed in an Illumina Novaseq 6000 DNA sequencer with a 150 bp paired-end sequencing strategy. The quality of raw Illumina reads was checked using the FastQC tool (V. 0.12.0) (https://www.bioinformatics.babraham.ac.uk/projects/fastqc/). Low-quality sequences with a Phred quality score (Q) below 20 and the Illumina adaptors were removed using TrimGalore (https://github.com/FelixKrueger/TrimGalore). Subsequently, reads were merged and *de novo* assembled using Trinity (2.9.0) [22], in single-stranded F mode, and ABySS (2.3.1) [23] with k values ranging from 25 to 95, with increments of 10. The final assemblies were merged, and sequences sharing at least 95% identity were consolidate using the CD-HIT (V4.7) tool [24]. The DNA coding sequences (CDS) with an open reading frame (ORF) of at least 150 nucleotides were extracted based on BLASTp results from several databases, including a subset of the non-redundant protein database containing all sequences belonging to the “chelicerata” taxonomic group, the non-redundant transcriptome shotgun assembly (TSA-nr), and Refseq-invertebrate. The CDS were extracted if they covered at least 70% of a matching protein. Additionally, all ORFs starting with a methionine and with a length of at least 40 amino acids were subjected to the SignalP tool (V3.0). Sequences with a putative signal peptide were mapped to the ORFs, and the most 5’ methionine was selected as the starting point of the transcript [25]. Relative quantification of each CDS was performed by mapping the trimmed Illumina reads to the final set of CDS using RSEM (V1.3.3) [26] and CDS with a TPM ≥ 5 in at least one biological condition was used as initial cut-off and selected for downstream analysis. Functional annotation of the selected CDS was carried out using an *in-house* program which is available upon request from Dr. Ribeiro (jribeiro@nih.gov). This program scanned a vocabulary of approximately 450 words and their order of appearance in the protein matches obtained from BLASTp/RPS-BLAST against various databases, including the TSA-nr, a subset from the NCBI Non-Redundant protein database containing all sequences belonging to the “chelicerata” taxonomic group, Refseq-invertebrate, Refseq-vertebrate, Refseq-protozoa, UNIPROTKB, CDD, SMART, MEROPS, and PFAM. All reference databases were obtained in August 2023. This annotation process included percent identities and coverage information [27]. The final annotated CDS are available for download as a hyperlinked Excel file (Supplementary file 1). Transcriptome completeness was evaluated using the Benchmarking Universal Single-Copy Orthologs (BUSCO) (V4.1.3) utilizing the Arachnida database as reference [28].

### Statistical analysis

The multidimensional plot and the pairwise differential expression analysis were carried out with the edgeR package [29] for R [30]. For the differential expression analysis, the raw read counts of each transcript was used as input for edgeR, followed the standard TMM-normalization. Statistical significance was considered when LogFoldChange (LogFC) higher than 2 or lesser than − 2, alongside a false discovery rate (FDR) less than 0.05 were obtained. The heatmap plot was generated with the pheatmap package using the TPM values and the volcano plots were generated with the ggplot2 package for R. Unsupervised clustering of the filtered CDS were performed with the Expander tool using the CLICK method [31]. Ortholog detection was determined by the reciprocal smallest distance (RSD) method [32], run with a coverage ≥ 80% and an e-value ≤ 0.1.

## Results

### The temporal transcriptome landscape of *A. Americanum* midgut as feeding progress

**Fig. 1:**
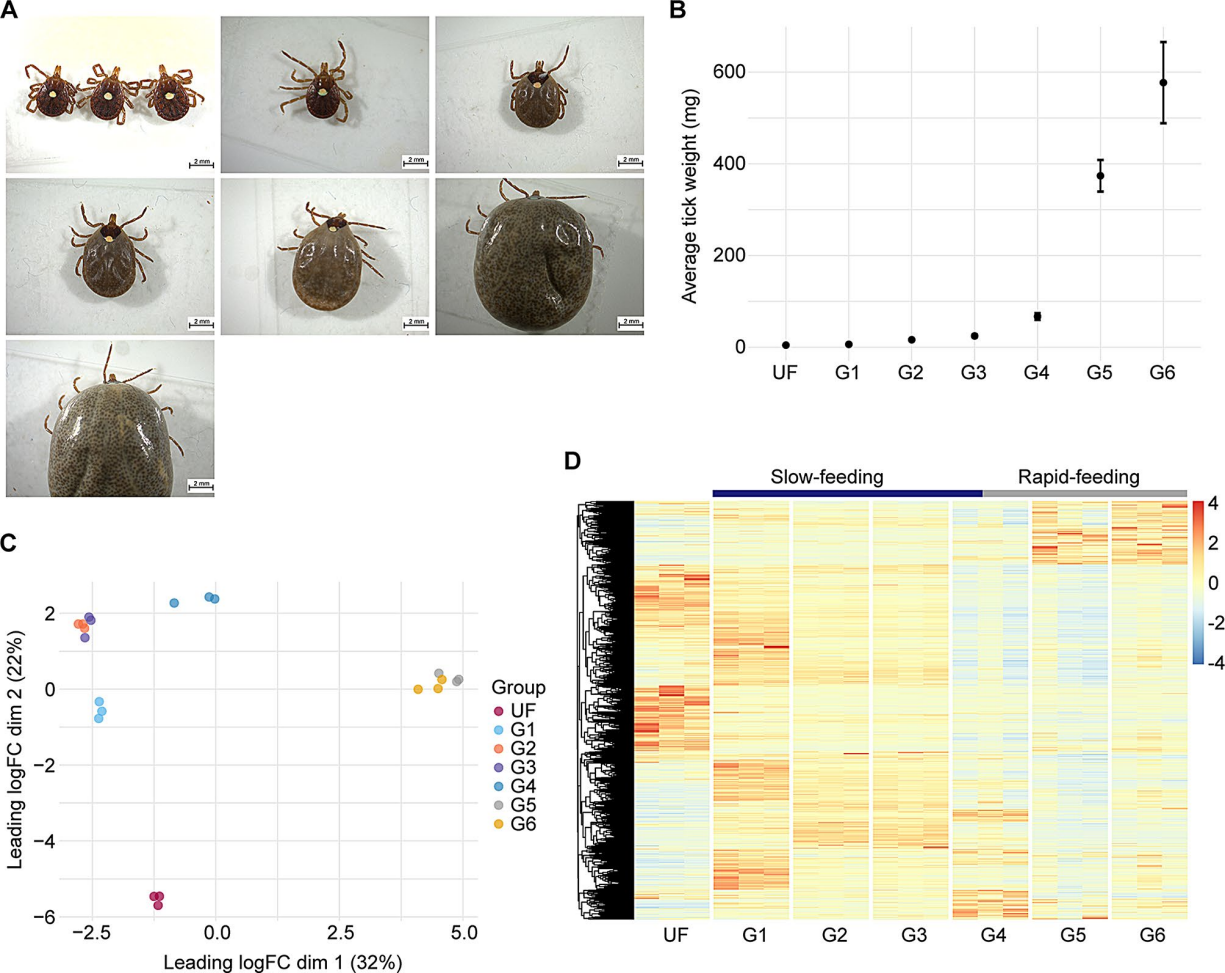
Overview of the transcriptome profile of *Amblyomma americanum* midgut at different feeding stages. (**A**) Representative images of *A. americanum* adult females collected at different feeding stages and their (**B**) average weight (± standard deviation of the mean). (**C**) Multidimension plot of the transcripts identified in *A. americanum* midgut with TPM ≥ 5 in at least one of the biological conditions. (**D**) Heatmap plot of the normalized TPM values of each transcript with TPM ≥ 5 identified in *A. americanum* midgut at each feeding stage

The Illumina-based RNA-sequencing of the 21 libraries from *A. americanum* midgut at sequential feeding stages (Fig. 1A and B) resulted in 1,068,002,018 high-quality reads. Following our *de novo* assembly and CDS extraction pipeline, we obtained a total of 127,756 putative CDS. When aligning the trimmed library reads to the predicted transcripts, we observed similar mapping rates across all biological conditions (49.5% ± 2.0%, Supplementary Table 1). For downstream analysis, we selected the CDS that exhibited a minimum TPM value of 5 in at least one biological condition, resulting in a final set of 15,599 CDS. Lastly, to assess the overall quality of our dataset, we employed the BUSCO, which indicated similar levels of completeness across all samples (59.9% ± 5.3%, Supplementary Table 2).

Dimensional analysis based on the TPM values from the final 15,599 CDS revealed that all biological replicates clustered within their respective biological conditions, without any notable outliers (Fig. 1C). Additionally, our samples formed four distinct groups corresponding to UF, slow-feeding (G1 – G3), a transitional stage from slow- to rapid-feeding (G4), and rapid-feeding (G5 and G6) phases. A similar pattern was evident when we generated a heatmap plot of the transcripts (Fig. 1D).

**Fig. 2:**
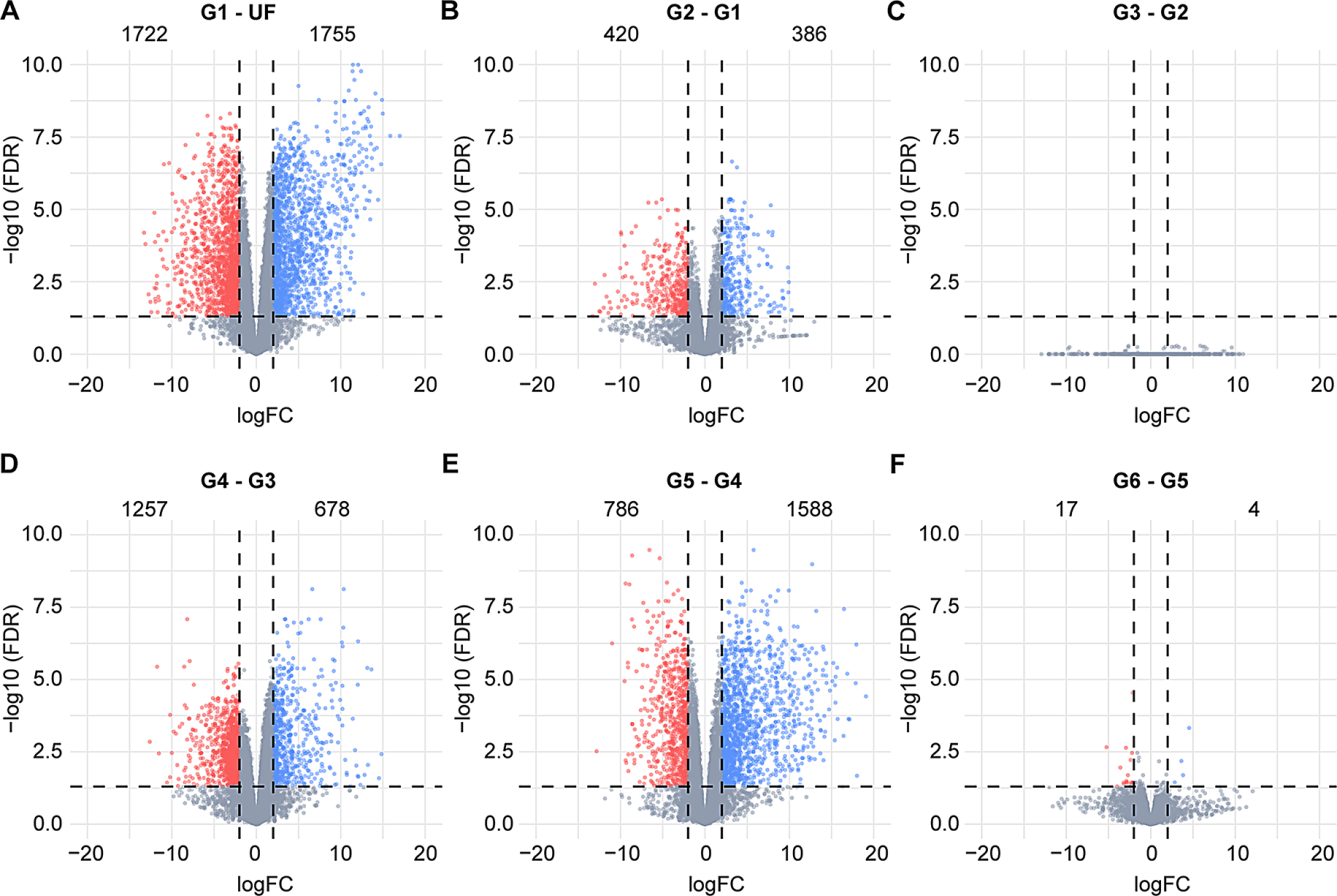
Volcano plots showing the differentially expressed transcripts obtained from the pairwise comparisons between the groups (**A**) G1 and unfed (UF), (**B**) G2 and G1, (**C**) G3 and G2, (**D**) G4 and G3, (**E**) G5 and G4, and (**F**) G6 and G5. The groups G1 to G6 represents ticks in different feeding stages that were group by their average weight; (G1) 6.4 ± 0.60 mg, (G2) 16.4 ± 1.82 mg, (G3) 24.7 ± 3.24 mg, (G4) 67.2 ± 7.30 mg, (G5) 373.9 ± 34.48 mg and (G6) 577.0 ± 88.50 mg. Statistical difference was considered when a transcript presented a LogFC higher than 2 or lesser than − 2 (vertical dotted lines), alongside a false discovery rate (FDR) ≤ 0.05 (horizontal dotted lines). Upregulated transcripts are shown as blue dots, downregulated transcripts are shown as red dots and gray dots represents transcripts that were not considered differentially expressed. Numbers inside each plot indicates de number of transcripts differentially expressed

To gain a comprehensive understanding of the dynamic transcriptional changes occurring in *A. americanum* midgut during blood acquisition, we performed a pairwise differential expression analysis for each feeding stage compared to the preceding stage (Fig. 2). The most significant transcriptional change was observed during the transition from UF to the initial feeding stage (G1), with 3,477 differentially expressed transcripts. During the slow-feeding stage, we noted a moderate number of transcripts displaying differential expression, particularly in the early stages (G2 – G1). Interestingly, despite a 50% increase in weight, there were no discernible differences between the midguts of ticks in the G2 (16.4 mg) and those in the G3 (24.7 mg). The G4 – G3 and G4 – G5 comparisons exhibited 1,935 and 2,374 modulated transcripts, respectively. Interestingly, almost no differences were found between ticks within the rapid-feeding phase (G6 – G5).

In parallel to the differential expression analysis, we also conducted an unsupervised clustering of the transcripts based on their TPM values, resulting in the categorization of the putative CDS into six primary patterns (Supplementary Fig. 1). Cluster 1 comprised 4,466 transcripts predominantly present in the UF stage. Cluster 2 encompassed, 4,398 CDS, representing sequences highly abundant in the G1 stage, signifying genes promptly induced upon initial contact with host blood. Congruent with the dimensional analysis and heatmap plots, cluster 3 (2,186 CDS) corresponded to transcripts exhibiting high abundance throughout the tick’s slow-feeding phase. While clusters 4 to 6 consisted of CDS mainly abundant during the rapid-feeding phase, but displaying distinct transcriptional patterns.

### Functional annotation of *A. americanum* midgut transcripts through feeding progression

**Fig. 3:**
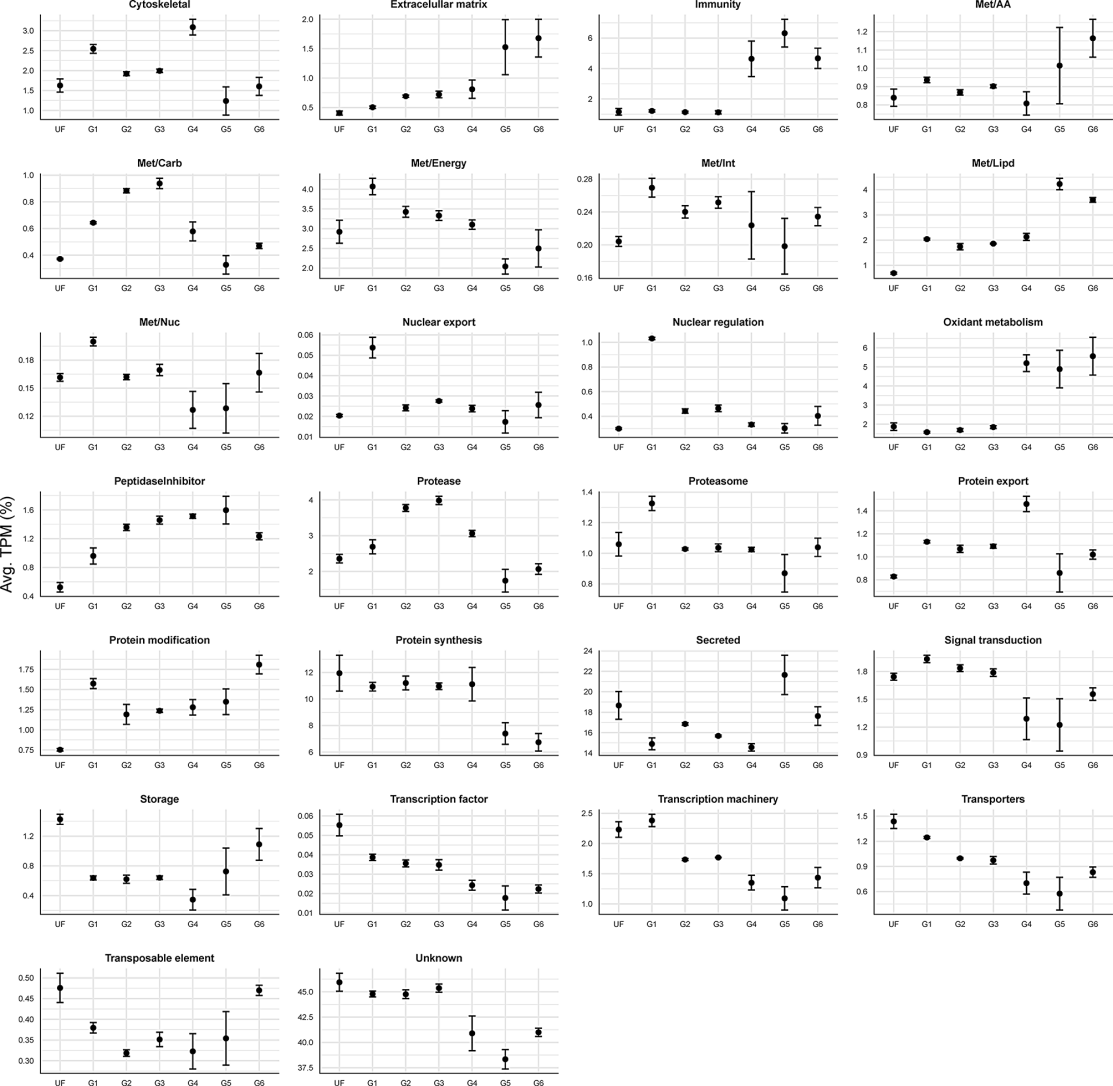
Relative quantification of the 26 functional classes over the different feeding stages of *A. americanum* midgut. Dots represent the average TPM (%) of each class at each biological condition. The error bars represent the standard deviation of the mean

To gain a deeper understanding of *A. americanum* midgut physiology during the feeding process, we systematically categorized the 15,999 putative CDS into 26 functional groups. Inspection of these functional classes across different feeding stages provided an overview of the temporal organization of various metabolic processes (Fig. 3). The resulting set of CDS, along with their functional annotation, is available for download in a Windows-compatible hyperlinked Excel file (Supplementary file 1).

It is important to mention that our *in-house* classification approach included the “unknown” class. This class encompass transcripts that bear resemblance to deposited sequences of unknown function or exhibit negligible similarities to previously deposited sequences, and thus can be considered potential novel sequences. In the current dataset, the “unknown” functional group was found to be the most abundant in all biological groups, accounting for 38.3–45.9% of the total TPM (Fig. 3). CDS annotated as “secreted” constituted the second most abundant class, accounting for 14.6–21.6% of all quantified CDS (Fig. 3). This class contains transcripts from different protein families containing a putative signal peptide.

Aside from their abundance, some functional classes presented a very clear transcriptional pattern as feeding progressed. Specifically, the “immunity” class, which includes multiple transcripts encoding putative antimicrobial peptides such as microplusin-like, lysozyme, and defensins, exhibits low TPM values throughout the unfed (UF) and slow-feeding stages (G1 – G3), representing 1.16% ± 0.03 of the total TPM (Fig. 3). However, the expression of this class largely increased during the transitional (G4: 4.6%) and the rapid-feeding stages (G5: 6.3% and G6: 4.7%). A nearly identical pattern was observed for the “oxidant metabolism” class, which encompasses putative catalases, superoxide dismutases, glutathione S-transferases, sulfotransferases, and thioredoxin-like proteins (Supplementary file 1).

### The midgut of unfed *A. americanum* adult females

**Fig. 4:**
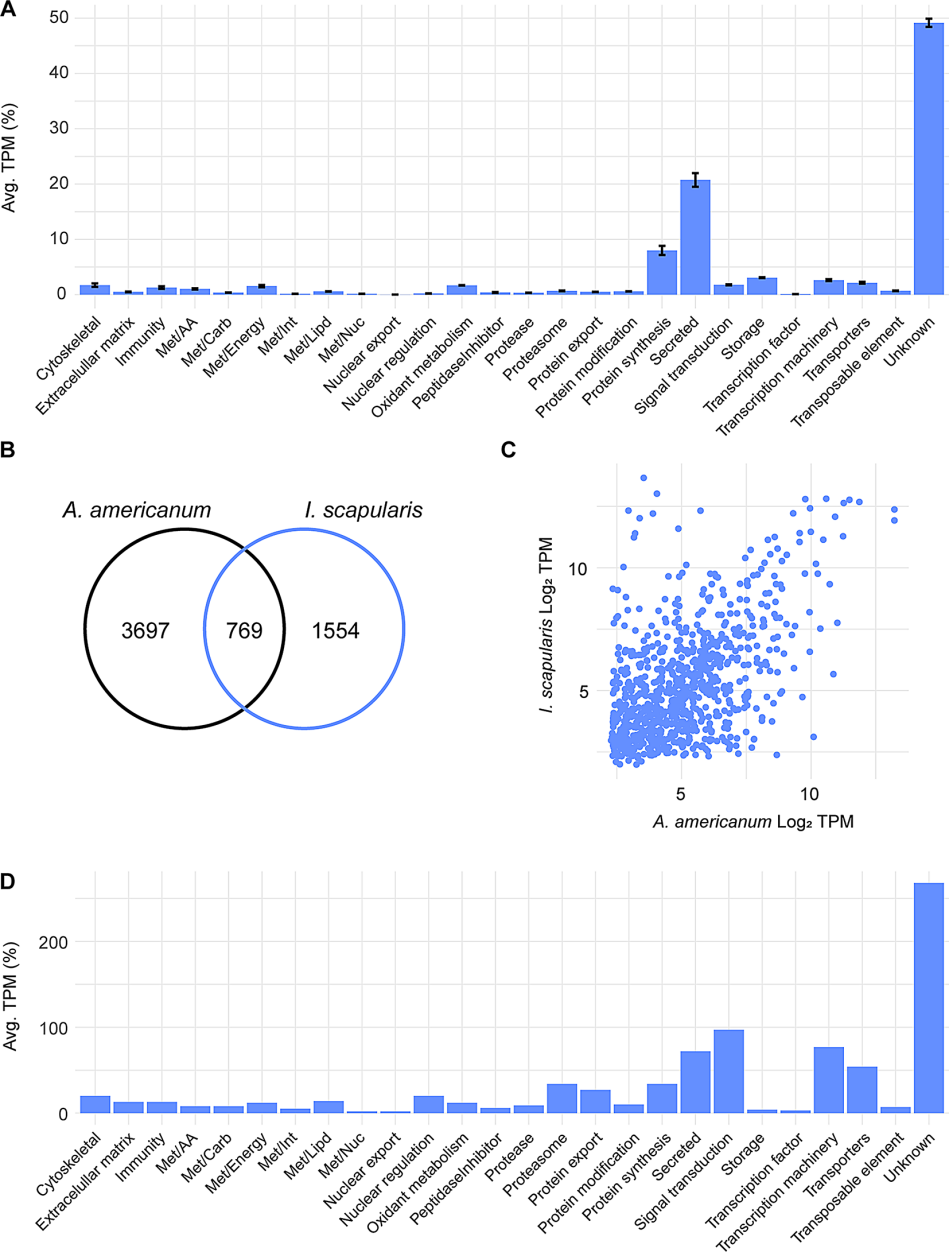
The transcriptional profile of unfed *A. americanum* adult female midgut. (**A**) Functional classification of 4,466 CDS abundant in the midgut of unfed ticks. Bars represent the average transcript per million (TPM) of each class, the error bars represent the standard deviation of the mean. Comparison between the abundant transcripts found in the midgut of unfed *A. americanum* and *I. scapularis*. (**B**) Venn diagram represent the number of transcripts unique and orthologous between both ticks. (**C**) Scatter plot of the Log_2_TPM of the 769 orthologous transcripts in the midgut of unfed adult females. (**D**) Functional classification of the shared transcripts between the unfed midguts of *A. americanum* and *I. scapularis* adult females. Bars represent the number of coding sequences (CDS) identified within each class

To characterize the transcriptional profile of the midgut of unfed ticks, we opted to focus on the 4,466 transcripts that were notably abundant in this stage by our unsupervised clustering analysis (Supplementary Fig. 1, cluster 1). Based on their TPM values, we found that the predominant functional classes within this cluster were “unknown” (49.1%), “secreted” (20.7%), and “protein synthesis” (8.0%) classes (Fig. 4A).

When comparing the transcriptome of unfed *A. americanum* adult females with that of unfed *I. scapularis* adult females [20], we found that *A. americanum* ticks exhibits a greater number of enriched CDS during the unfed stage compared to *I. scapularis* (Fig. 4B). Upon scrutinizing conserved sequences between these ticks at this stage, only 769 CDS met our criteria by RSD analysis (Fig. 4B and Supplementary file 1). It is important to note that these 769 CDS displayed comparable TPM levels in both tick species (Fig. 4C), exhibiting a Pearson correlation coefficient of 0.52 (*p* < 0.05).

Further exploration of the functional classification of the 769 shared CDS revealed that the majority of them were classified within the “unknown” (268 CDS), “signal transduction” (97 CDS), “transcription machinery” (77 CDS), and “secreted” (72 CDS) classes (Fig. 4D).

### The midgut of slow-feeding *A. americanum* adult females

As mentioned before, we observed a conserved transcriptional profile during the slow-feeding stage of *A. americanum* adult females, wherein most of the initial changes were induced by the incoming blood meal (Fig. 2). When exploring the differentially expressed transcripts between the G1 and unfed groups (Table 1), we observed an overall increase of the “lipid metabolism (Met/Lipd)”, “protein modification” and “peptidase inhibitors” functional classes, while the “transcription factor”, “protein synthesis” and “transporters” were the most downregulated classes.

The transcripts belonging to the “peptidase inhibitor” class displayed elevated TPM values in the G1 group, exhibiting a 2.93-fold increase compared to the unfed group (Table 1). Within this class, we identified various serine peptidase inhibitors from the serpin, Kunitz-type, and trypsin inhibitor-like (TIL) subfamilies, as well as cystatins, which are tight-binding cysteine peptidase inhibitors. Notably, two CDSs accounted for 89.7% of the total TPM of this class within the G1 group (Supplementary file 1). The CDS Amseq_222115 encoded a putative type-2 cystatin, showing the highest modulation based on TPM values (UF_TPM_ = 528 and G1_TPM_ = 3,906). While seqSigP-25462, encoding a putative boophilin-like protein, emerged as the second most abundant peptidase inhibitor transcript within the G1 group (TPM = 1190.69). Although other peptidase inhibitors were found differentially expressed between G2 and G1 groups, their overall TPM values were low (Table 2), underscoring the significance of Amseq_222115 and seqSigP-25462 as the primary modulated peptidase inhibitors during the slow-feeding of *A. americanum* adult females.

**Table 1:** Functional classification of the differentially expressed transcripts between the midguts of the G1 and unfed *A. americanum* adult females

Class	No. of transcripts		UF TPM	G1 TPM	G1/UF TPM
	Down	Up			
Nuclear regulation	6	35	470.48	4056.29	8.62
Met/Lipd	19	40	1166.13	10028.99	8.60
Nuclear export	0	9	15.33	115.21	7.52
Protein modification	7	27	1828.74	6849.27	3.75
Extracellular matrix	5	19	238.04	818.35	3.44
Peptidase inhibitors	21	13	1940.14	5677.87	2.93
Met/Carb	8	18	257.68	751.13	2.92
Met/Nuc	3	8	124.21	240.69	1.94
Cytoskeletal	14	19	4605.97	8456.49	1.84
Peptidase	9	16	793.32	1386.39	1.75
Immunity	24	22	6179.63	6068.01	0.98
Transcription machinery	19	19	1608.80	1475.96	0.92
Protein export	15	11	895.99	755.00	0.84
Met/AA	6	13	538.60	447.54	0.83
Met/Energy	27	32	2612.86	2089.25	0.80
Oxidant metabolism	48	36	6304.54	4801.59	0.76
Storage	3	4	2064.73	1422.31	0.69
Signal transduction	40	38	1908.36	1246.09	0.65
Unknown	956	975	107006.75	59145.66	0.55
Proteasome	15	10	1072.43	526.26	0.49
Secreted	346	325	75949.14	36603.59	0.48
Met/Int	3	6	479.61	226.21	0.47
Transposable element	56	11	1108.11	463.90	0.42
Transporters	59	40	4079.35	1626.14	0.40
Protein synthesis	12	9	5875.61	2149.11	0.37
Transcription factor	1	0	10.99	3.36	0.31

*TPM: Transcript Per Million

As the slow-feeding progresses, we observed further transcriptional changes within de midgut of *A. americanum* ticks. Among the 806 differentially expressed transcripts identified in the G2 – G1 comparison, transcripts classified within the “carbohydrate metabolism – Met/Carb”, “extracellular matrix” and “secreted” were found to be the most upregulated ones. While transcripts related to the “nucleotide metabolism – Met/Nuc” and “protein export” were found to be the most downregulated transcripts.

**Table 2:** Functional classification of the differentially expressed transcripts between the midguts of the G2 and G1 *A. americanum* adult females

Class	No. of transcripts		G1 TPM	G2 TPM	G2/G1 TPM
	Down	Up			
Met/Carb	0	5	317.85	1480.93	4.66
Extracellular matrix	3	7	138.97	504.00	3.63
Secreted	91	86	8596.11	30810.94	3.58
Storage	0	2	8.89	30.84	3.47
Protein synthesis	0	1	1.95	6.43	3.30
Unknown	217	202	13263.17	39129.18	2.95
Oxidant metabolism	16	7	731.77	2025.74	2.77
Protease	5	4	917.89	2106.61	2.30
Signal transduction	12	6	401.03	782.21	1.95
Met/AA	4	3	121.04	179.97	1.49
Transcription machinery	2	2	48.48	62.55	1.29
Cytoskeletal	3	1	55.34	62.86	1.14
Transposable element	4	8	61.37	51.40	0.84
Met/Lipd	13	10	6322.50	3808.34	0.60
Immunity	8	4	1626.83	941.31	0.58
Met/Energy	5	6	185.36	90.33	0.49
Peptidase inhibitor	12	4	439.63	188.44	0.43
Protein modification	2	1	46.36	15.93	0.34
Transporters	14	4	105.39	34.47	0.33
Proteasome	1	0	13.80	2.54	0.18
Nuclear regulation	1	0	1253.03	207.92	0.17
Met/Int	1	0	24.99	3.63	0.15
Met/Nuc	2	0	34.72	3.80	0.11
Protein export	3	0	75.82	6.08	0.08

*TPM: Transcript Per Million

**Fig. 5:**
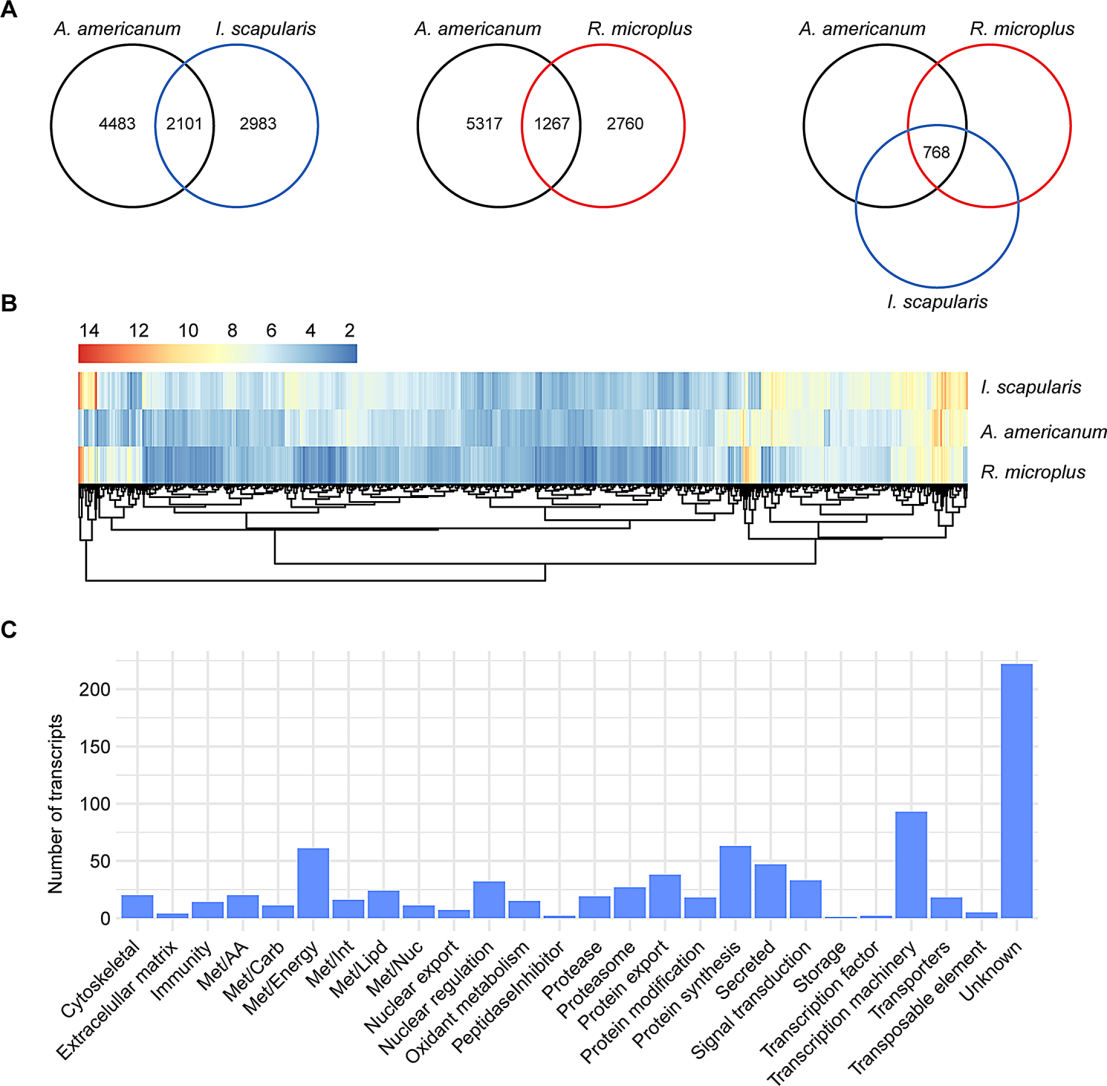
Comparison of the transcriptional profile of the midgut of slow-feeding *A. americanum*, *I. scapularis*, and *R. microplus*. (**A**) Venn diagrams displaying the number of unique and orthologous transcripts across the different tick species. (**B**) Heatmap plot based on the Log_2_TPM of the 768 orthologous transcripts and their (**C**) functional classification

By comparing the slow-feeding transcriptional profile of *A. americanum* with those observed in *I. scapularis* [20] and *R. microplus* [21], we identified 2,101 and 1,267 shared orthologs, respectively (Fig. 5A). Of those, 768 were common across the three species. Upon evaluating the overall abundance of these shared transcripts within the midgut of each tick species, we found that most exhibited moderate levels of TPM, with a majority presenting Log_2_TPM values ranging from 2 to 10 (Supplementary Fig. 2A). Additionally, when comparing the abundance of these shared transcripts across the three tick species, we observed comparable levels (Fig. 5B and Supplementary Fig. 2B).

Further exploration of the functional annotation of the 768 shared transcripts revealed that most were classified as “unknown” (222 CDS). The remaining transcripts were categorized into functional classes mainly related to housekeeping process, including “transcription machinery” (93 CDS), “protein synthesis” (63 CDS) and “energetic metabolism – Met/Energy” (61 CDS) (Fig. 5C).

### The rapid-feeding midgut of *A. americanum* adult females

The transition from the slow- to rapid-feeding stage represents an important step on the tick life cycle. Practically, collecting ticks in this specific phase is challenging due to the short timeframe of this transition. In the current dataset, we observed a high number of differentially expressed transcripts in the G4 – G3 and G5 – G4 comparisons. Therefore, we understand that G4 ticks represent the beginning of the transition from slow- to rapid-feeding and have included this information in this section.

In our G4 – G3 differential expression analysis, we uncovered a substantial number of differentially expressed transcripts (Table 3). The classes exhibiting the most pronounced changes include “immunity” (7.28-fold), “oxidant metabolism” (6.07-fold) and “peptidase inhibitors” (4.63-fold). Within the “immunity” class, we observed an upregulation of numerous transcripts encoding Toll-like receptors, microplusin-like, and lysozyme-like proteins (Supplementary file 1). This elevation of the number of immune-related transcripts persisted throughout the rapid-feeding stage of the adult female (Fig. 3; Table 4).

**Table 3:** Functional classification of the differentially expressed transcripts between the midguts of the G4 and G3 *A. americanum* adult females

Class	No. of transcripts		G3 TPM	G4 TPM	G4/G3 TPM
	Down	Up			
Immunity	10	11	4944.51	35993.81	7.28
Oxidant metabolism	18	23	5841.90	35485.70	6.07
Peptidase Inhibitor	2	14	1005.71	4655.89	4.63
Nuclear export	0	2	17.07	66.44	3.89
Protein export	15	14	1133.67	4007.39	3.53
Cytoskeletal	10	14	2394.23	7356.28	3.07
Transposable element	22	13	505.5167	1340.037	2.65
Met/Lipd	24	12	4444.32	11667.91	2.63
Extracellular matrix	12	4	632.44	1649.09	2.61
Secreted	209	136	16503.82	40711.89	2.47
Protease	7	4	188.53	457.10	2.42
Protein modification	7	4	459.26	1104.46	2.40
Met/AA	7	13	225.45	540.82	2.40
Met/Energy	13	7	1023.75	1929.56	1.88
Unknown	715	356	24839.76	44302.71	1.78
Met/Int	4	3	150.58	213.98	1.42
Met/Carb	9	7	314.91	440.33	1.40
Signal transduction	61	12	735.47	585.38	0.80
Transporters	52	15	1025.65	807.87	0.79
Transcription machinery	36	5	369.54	270.60	0.73
Nuclear regulation	6	2	34.77	17.14	0.49
Proteasome	5	3	53.58	22.56	0.42
Met/Nuc	4	1	116.74	17.06	0.15
Protein synthesis	5	0	40.94	4.97	0.12
Transcription factor	3	0	37.82	4.03	0.11
Storage	3	0	32.86	1.84	0.06

*TPM: Transcript Per Million

The comparison between G5 and G4 ticks represents the end of transition from slow- to rapid-feeding adult females. In this final feeding stage, the adult females ingested a vast amount of host blood within a relatively short timeframe, typically, lasting between 12 and 24 h. This stage is commonly referred to as the “big sip”, marked by a significant increase in the tick’s body size and mass (Fig. 1A and B). By the conclusion of this stage, the tick naturally detaches from its host. This comparison yielded the second largest number of differentially expressed transcripts (786 downregulated and 1,588 upregulated) within our dataset. Furthermore, when considering the overall TPM values (> 5,000) of the modulated functional classes, we found the “lipid metabolism – Met/Lipd” (16.38-fold), “extracellular matrix” (10.35-fold) and “immunity” (3.65-fold) classes to be the most abundant ones (Table 4).

**Table 4:** Functional classification of the differentially expressed transcripts between the midguts of the G5 and G4 *A. americanum* adult females

Class	No. of transcripts		G4 TPM	G5 TPM	G5/G4 TPM
	Down	Up			
Met/Lipd	14	33	1023.98	16770.25	16.38
Transcription factor	0	2	1.65	20.39	12.33
Storage	2	3	27.09	322.79	11.92
Extracellular matrix	18	11	761.64	7883.53	10.35
Protein synthesis	0	4	2.83	16.86	5.95
Proteasome	10	2	37.17	215.04	5.79
Met/AA	12	13	736.23	3964.92	5.39
Protein modification	11	10	1025.77	4808.10	4.69
Nuclear regulation	0	2	1.88	7.69	4.10
Immunity	10	24	1425.33	5198.91	3.65
Secreted	157	339	35916.35	105819.84	2.95
Met/Int	3	5	129.85	350.71	2.70
Met/Energy	8	30	1202.11	2214.43	1.84
Unknown	417	831	56853.95	102607.78	1.80
Transcription machinery	4	13	250.08	349.76	1.40
Met/Nuc	3	6	77.92	106.44	1.37
Signal transduction	17	57	910.03	1204.50	1.32
Oxidant metabolism	27	36	1613.26	1946.96	1.21
Transposable element	8	30	1330.62	1585.53	1.19
Transporters	22	49	893.36	1035.69	1.16
Peptidase Inhibitor	3	24	10639.80	9588.33	0.90
Met/Carb	9	8	319.46	150.12	0.47
Protease	17	28	17814.66	5877.39	0.33
Protein export	9	12	1826.01	455.23	0.25
Cytoskeletal	12	8	3697.19	881.24	0.24
Nuclear export	1	0	24.28	5.45	0.22

*TPM: Transcript Per Million

Despite the substantial difference in weight between G5 (373.9 ± 32.5 mg) and G6 (577.0 ± 83.4 mg), our differential expression analysis revealed a modest distinction between these two groups (Fig. 3), involving a total of 21 modulated transcripts (17 downregulated and 4 upregulated).

## Discussion

The current longitudinal transcriptome analysis from the midguts of *A. americanum* female ticks highlights the dynamic changes that take place within this tissue as feeding progress. Here, we opted to group our ticks by their weight in order to better represent and characterize the transcriptional profiles of the slow- and rapid-feeding stages. As demonstrated by dimension analysis and heatmap plots (Fig. 1C and D), this approach proved to be a good method for estimation of the tick feeding stage, since all our biological replicates were found with a high degree of similarities. Furthermore, based on our clustering and differential expression analysis, we identified four main transcriptional profiles that can be attributed to unfed, slow-feeding, the transition from the slow- to the rapid-feeding stage and the rapid-feeding stages.

As reported for other tick species [13, 14, 20, 21], the onset of feeding and the initial contact of the tick midgut with the host blood triggers a dramatic change in the transcriptional profile of *A. americanum* adult females, in which the highest number of differentially expressed transcripts was found. Remarkably, despite the slow-feeding stage spanning multiple days, the limited number of differentially expressed transcripts observed within this stage indicates the presence of a somewhat conserved transcriptional profile throughout the slow-feeding period. However, it is important to recognize the need of proteomic studies to confirm if the protein contents of the tick midgut are also conserved.

Considering the high number of differentially expressed transcripts in the G4 – G3 and G4 – G5 comparisons, as well as the significant difference in the average weights among these groups, it appears that the G4 group represents a transitional stage between slow- and rapid-feeding phases. Interestingly, ticks within the G4 group exhibits an average weight of approximately 14.3-fold the average weight of unfed ticks, which is near the determined “critical weight” of *A. americanum* [33]. Given that the weight difference between G5 and G4 was more than five-fold, future studies aimed at collecting ticks within the 50–380 mg weight range will likely provide a finer resolution of this transition, which represents a key point in the tick life cycle.

Functionally, the high prevalence of the “unknown” class across all our biological groups highlights the existing knowledge gap regarding the composition and function of potential proteins present in the tick midgut, emphasizing the need of further studies focused on this organ. The second most abundant overall functional class was the “secreted”, which encompasses transcripts from different protein families containing a putative secretion signal. Such sequences are commonly reported in the sialome of blood-feeding arthropods and includes sequences bearing similarities to antigen 5-like proteins, lipocalins, lipases, hormone-binding proteins, and mucins [34, 35].

Our analysis of the functional classes across different feeding stages revealed some intriguing patterns. Specifically, the “immunity” class peaked during the transition from the slow- to the rapid-feeding stage. During this phase, the mated adult female ingests a substantial volume of blood in a relatively short timeframe [8]. It is likely that the rapid accumulation of the blood meal within the midgut lumen creates a favorable environment for the proliferation of microbes that could be detrimental to the tick if not properly controlled. It is worth noting that a similar pattern of immune-related transcripts was observed in the midgut of the cattle tick *R. microplus* [21]. In contrast, *I. scapularis* adult females showed a significant increase in immune-related transcripts at the onset of the slow-feeding phase [20].

A great effort has been made to characterize the immune response of ticks [36, 37]. A recent study focused on the early immune response of *I. ricinus* adult females to bacterial challenge revealed the absence of upregulated immune-related transcripts in the midgut. However, proteomic analysis has identified several antimicrobial peptides (AMP) that are abundant regardless of microbial challenge, termed “guard AMPs” and suggested to act as the first line of defense within the tick midgut [38]. A second study focused on the effects of partially fed *R. rickettsii*-infected and non-infected midguts from *Amblyomma sculptum* and *Amblyomma aureolatum* demonstrated distinct transcriptional responses of immune-related sequences between the two tick species [39]. Together, the distinct temporal transcriptional responses of immune-related sequences across different tick species highlight the diverse strategies tick have evolved to manage microbial proliferation in their midguts. Furthermore, these differences likely play a significant role their susceptibility to pathogens, and therefore in their vector competency.

Similar to the “immunity” class, transcripts related to “oxidative metabolism” also peaked in the midgut of *A. americanum* adult females from the G4 group. When host blood enters the midgut lumen, red blood cells are lysed through a yet-to-be-discovered mechanism releasing hemoglobin [40]. The breakdown of hemoglobin subsequently results in the release of significant amounts of heme, which is prone to causing oxidative damage [41]. To mitigate this potential damage, ticks have evolved several mechanisms, including the aggregation of heme into specialized organelles known as “hemosome” [42] and the production of antioxidant enzymes such as superoxide dismutases, catalases, and glutathione S-transferases.

Interestingly, it seems that ticks have lost the ability to synthesize heme, as key enzymes in the heme biosynthesis pathway are missing from their genomes [43, 44]. Consequently, the incorporation of dietary heme into tick heme-proteins serves as a strategy for heme detoxification [45]. In this context, the specific upregulation of the “oxidant metabolism” class during the G4 stage may be seen as a controlled response to the accumulation of heme in the tick midgut. In contrast, transcripts associated with “oxidative metabolism” were abundant throughout the slow-feeding stage in both *R. microplus* and *I. scapularis* [20, 21], suggesting the presence of different, yet unknown, regulatory mechanism across different tick species.

It is worth noting that proteomic analysis of midguts from *I. ricinus* nymphs at various feeding stages [46] revealed trends similar to the ones observed in our study. Specifically, the abundance of the “oxidant metabolism” class was low in unfed and slow-feeding nymphs but was upregulated in fully-fed nymphs. Similarly, the “proteases” and “protein synthesis” classes in *I. ricinus* nymphs showed patterns comparable to those in *A. americanum* adult females. These transcripts and proteins were prevalent in unfed and slow-feeding ticks, with a significant decreased observed in rapidly-feeding and fully-fed ticks. These similarities suggests that certain midgut processes during feeding are conserved across nymphs and adult ticks. Given that targeting midgut proteins has proven effective for tick control [17, 47], identifying conserved targets across different tick species and life stages could be of particular interest for developing alternative tick control methods.

In unfed *A. americanum* adult females, we found that 4,466 transcripts were prevalent in this stage. Specifically, a putative ferritin (Amseq_49087), with an average TPM of 9,522, was found among the most abundant transcripts in the midgut of unfed females. Currently, several ferritins have been identified and characterized across different tick species’ midguts [48–50]. These can be further classified into type 1 and type 2 ferritins. Type 1 ferritins are intracellular proteins primarily involved in iron storage, with their transcription regulated by the presence of an iron-responsive element (IRE) in their 5’-UTR [51, 52]. Conversely, type 2 ferritins lack the IRE domain but possess a putative signal peptide. Further studies indicate that type 2 ferritins are predominantly produced in the tick midgut, facilitating the transport of iron from the midgut to other tissues [53]. Given their pivotal role in tick metabolism, these proteins have been suggested as promising targets for anti-tick control strategies [54]. Noteworthy is the Amseq_49087 mRNA, which displays an IRE domain within its 5’-UTR and exhibits substantial sequence homology with other type 1 ferritins from ticks (Supplementary Fig. 3). Although this transcript presents elevated TPM values within the midgut of unfed adult females, its expression remains relatively stable throughout the feeding phase, with TPM values oscillating between 1,147 and 3,633. This consistency suggests the presence and role of this ferritin throughout the entirety of the adult tick’s feeding cycle.

When comparing the transcriptome profiles of unfed *A. americanum* adult females with those of unfed *I. scapularis* adult females [20], we identified 769 putative transcripts that were conserved in both ticks and exhibited comparable TPM levels (Person correlation of 0.52, *p* < 0.05). This correlation suggests that these shared sequences are likely present in similar levels within the unfed midgut of both species. Supporting this hypothesis, we identified two type 1 ferritins from *I. scapularis* (XP_040076988.1 and XP_029846889.1) with TPM values of 5,304 and 3,890, respectively, ranking them among the most abundant transcripts in unfed ticks.

As mentioned before, the transition from unfed to slow-feeding ticks accounts for the highest number of differentially expressed transcripts in adult females [13, 14], and in *A. americanum* the most modulated functional class during this transition was the “lipid metabolism – Met/Lipd”. Currently, our understanding of how ticks metabolize lipids is limited, with only a few studies dedicated to this topic [55, 56]. In our current dataset, we identified 59 differentially expressed transcripts between the G1 and UF ticks (Table 1) that encode putative proteins related to lipid metabolism. Notably, the downregulated CDS included putative enzymes related to lipid catabolism, such as putative acyl-CoA synthetases, which play a crucial role in activating fatty acids prior to β-oxidation, and acyl-CoA oxidases, which catalyze the initial step of fatty acid β-oxidation. Additionally, several transcripts encoding putative triacylglycerol lipases were observed, involved in the hydrolysis of triacylglycerols to glycerol and fatty acids. Conversely, among the upregulated CDS, we identified transcripts for putative enzymes related to lipid biosynthesis. This included enoyl-CoA reductases, important in the synthesis of fatty acids, as well as fatty acid synthetases and sterol O-acyltransferases. The latter encompasses enzymes that convert saturated fatty acids into monosaturated fatty acids, serving as precursors for the synthesis of various lipids. This trend was also observed within the differentially expressed transcripts of the G2 – G1 comparison (Table 2), where additional triacylglycerol lipases and a putative carnitine O-palmitoyl transferase I, an enzyme related to the transport of long-chain fatty acids into the mitochondria for β-oxidation, were found downregulated.

Transmission electron microscopy of midguts from partially engorged *Amblyomma cajennense* adult females revealed the presence of lipid droplets within the digestive cells [9]. Although the biochemical characterization of how dietary lipids are digested, absorbed, and transported are still lacking in ticks, based on the transcriptional modulation of CDS encoding putative transcripts related to lipid metabolism, it appears that as the host blood enters the tick midgut and throughout the slow-feeding phase, there is a transcriptional change that switches the lipid metabolism landscape from a catabolic profile to an anabolic one, potentially resulting in the formation of lipid droplets. This observation aligns with tick feeding biology, as it has been suggested that during the slow-feeding phase, ticks extract oligonutrients and/or lipids from the blood, while eliminating excess or unnecessary components through saliva or fecal material [57].

Within the midgut of slow-feeding *A. americanum* females, we identified two peptidase inhibitors encoding cystatin and boophilin-like proteins, which accounted for the majority of this class. Currently, several midgut cystatins from ticks have been characterized and associated with the regulation of hemoglobin degradation [58–60], and potentially interacting with cysteine peptidases from tick-borne pathogens [61, 62]. The putative transcript Amseq_222115 exhibited high similarities with other type-2 cystatins that were found to be abundant during the slow-feeding phase of *I. scapularis* [20] and *R. microplus* [21] (Supplementary Fig. 4). This observation suggests a conserved role of cystatins within the tick midgut, acting as major regulators of endogenous cysteine peptidases during the slow-feeding stage. Boophilin is a serine peptidase inhibitor predominantly found in the midgut of the cattle tick *R. microplus* [63] that contains two Kunitz-type domains, in which the N-terminal domain can bind and inhibit thrombin in a non-canonical manner [64]. Furthermore, thrombin-bound boophilin retains the ability to inhibit other serine peptidases, suggesting independent functionality of both Kunitz-type domains. It is proposed that such serine peptidases inhibitors play a crucial role in preventing blood coagulation within the tick midgut, facilitating the accumulation, digestion, and absorption of the blood meal. Similar to boophilin, seqSigP-25462 also exhibits two Kunitz-type domains; however, it possesses two positively charged residues (Arg and Lys) at the P1 of each domain, instead of the Lys and Ala found in boophilin (Supplementary Fig. 5). This finding indicates that the two domains of seqSigP-25462 can potentially act as thrombin inhibitors, hence, it is likely that this Kunitz-type inhibitor plays a major role in blocking blood-clotting within the midgut of *A. americanum* adults during the slow-feeding stage.

An increasing number of transcripts encoding putative serine peptidases have been identified in the midgut of ticks at different feeding stages [20, 21], although their roles in midgut physiology remain unclear. The intracellular digestion of hemoglobin in the tick midgut has primarily been attributed to aspartic and cysteine peptidases [10]. In our current dataset, we have identified several conserved transcripts encoding serine peptidases from the S1A subfamily (Supplementary Fig. 6). Recent research has shown that trypsin-like proteolytic activity is absent in the midgut of partially fed *I. scapularis* adults but increases in post-detached ticks [65]. Similarly, in *Haemaphysalis longicornis* adult females, various serine peptidase-like proteins are present in the midgut of partially fed ticks [66, 67]. Furthermore, knockdown experiments targeting such transcripts resulted in reduced hemolysis in the tick midgut, suggesting their involvement in blood digestion. The presence of serine peptidases, particularly abundant during the slow-feeding stage across different tick species, suggests a conserved and specific role of these enzymes. One possibility is the involvement of such peptidases in cellular signaling, akin to the mechanism observed via protease-activated receptors (PARs) in vertebrates. However, additional studies are needed to ascertain the presence of such receptors in invertebrates. Future studies aimed at the characterization of these serine peptidase-like proteins and their regulation will enhance our current understanding of blood meal digestion in ticks [10].

In the current dataset, we observed a high number of differentially expressed transcripts in the G4 – G3 and G5 – G4 comparisons. Therefore, we understand that G4 ticks represent the beginning of the transition from slow- to rapid-feeding. As discussed previously, this transition was characterized by a significant surge in the “immunity” and “oxidant metabolism” classes, which are likely linked to vast amount of blood ingested by the mated females. Within G5 ticks, we found that the transcript Amseq_83561, classified in the “lipid metabolism” class, accounted for 56% of the total TPM of this class (Supplementary file 1). This particular transcript encodes a putative farnesoic acid o-methyltransferases and is presently truncated in its 5’ portion. In insects, this enzyme catalyzes the methylation of farnesoic acid, converting it into methyl farnesoate - a biologically active form of juvenile hormone known for its involvement in various aspects of insect physiology, including molting, reproduction, and vitellogenesis [68]. In ticks, studies have suggested that juvenile hormone is not produced [69] and it has no impact on tick vitellogenesis [70, 71]. Instead, ticks appear to regulate vitellogenesis through 20-hydroxyecdysone signaling [72, 73]. Notably, enzymes belonging to the mevalonate pathway and the juvenile hormone branch have been identified in ticks [74], suggesting their potential in synthesize juvenile hormone precursors. Currently, their specific roles in tick physiology remain unknown.

Lastly, although a significant difference in the average weight of G5 and G6 ticks was found, only a handful of differentially expressed transcripts was observed. Corroborating to the notion that rapid-feeding *A. americanum* adult females have a consistent transcriptional profile during this stage. By the end of this stage the adult female naturally detaches from its host. In our previous longitudinal transcriptome studies of *I. scapularis* and *R. microplus*, we noted a considerable number of differentially expressed transcripts 24 h post-detachment [20, 21]. However, in the current study, our focus was directed towards exploring the unfed, slow- and rapid-feeding stages of *A. americanum*. We recognize the need for an additional study targeting latter time points to provide a more comprehensive understanding of the transcriptional changes during the feeding cycle of *A. americanum* adult females.

## Conclusion

The feeding process of *A. americanum* adult female ticks is accompanied by a significant morphological transformation characterized by a substantial increase in weight. In this study, we illustrate that the midgut across various feeding stages (unfed, slow-, and rapid-feeding) exhibits distinctive transcriptional profiles, shedding light not only on its composition but also on the presence of precise transcriptional regulatory mechanisms. The dataset presented here serves as a foundational steppingstone for future research endeavors directed at gaining a deeper understanding of tick midgut physiology.

## Electronic supplementary material

Below is the link to the electronic supplementary material.

Supplementary Material 1

Supplementary Material 2

## Data Availability

The transcriptome data was deposited to the National Center for Biotechnology Information (NCBI) under BioProject PRJNA1083553 and BioSample accessions SAMN40267879 - SAMN40267899. The raw reads were deposited to the Short Reads Archive of the NCBI under accessions SRR28230933 - SRR28230953. This transcriptome shotgun assembly was deposited at DDBJ/ENA/GenBank under the accession GKSP00000000. The version described in this paper is the first version, GKSP01000000. All supplementary files can be downloaded as a single compressed (.zip) file from the link: https://proj-bip-prod-publicread.s3.amazonaws.com/transcriptome/Aamericanum_Mg_2024/Aamericanum_Mg_SupData.zip.
