# Supplementary material for: Exploring the longitudinal expression dynamics of midguts in adult female Amblyomma americanum ticks
Source: BMC Genomics. 2024 Oct 24;25:996. doi: 10.1186/s12864-024-10905-y (PMC11515579; doi:10.1186/s12864-024-10905-y)
Supplement: Supplementary file 2 — Supplementary Material 2 [file 12864_2024_10905_MOESM2_ESM.pdf]

Supplementary table 1: Mapping rates of the trimmed Illumina reads to the putative CDS obtained from the *de novo* assembly of *A. americanum* midgut transcripts.

Sample	Reads not aligned	Reads aligned	Total reads	Reads aligned (%)
UF.A	28,710,730	26,258,152	54,968,882	47.76
UF.B	30,391,122	25,576,442	55,967,564	45.69
UF.C	27,004,578	23,953,610	50,958,188	47.00
G1.A	22,386,518	20,958,188	43,344,706	48.35
G1.B	23,009,394	21,885,922	44,895,316	48.74
G1.C	24,845,561	21,745,039	46,590,600	46.67
G2.A	36,856,265	33,889,303	70,745,568	47.90
G2.B	26,483,883	26,363,995	52,847,878	49.88
G2.C	27,136,423	25,931,353	53,067,776	48.86
G3.A	21,878,033	20,861,427	42,739,460	48.81
G3.B	20,460,881	19,995,267	40,456,148	49.42
G3.C	21,699,051	20,822,443	42,521,494	48.96
G4.A	19,853,516	22,085,700	41,939,216	52.66
G4.B	25,514,147	27,272,865	52,787,012	51.66
G4.C	22,419,683	26,471,151	48,890,834	54.14
G5.A	27,697,473	28,030,731	55,728,204	50.29
G5.B	24,356,043	26,071,825	50,427,868	51.70
G5.C	29,489,917	30,514,159	60,004,076	50.85
G6.A	25,198,802	25,094,764	50,293,566	49.89
G6.B	30,571,143	29,736,863	60,308,006	49.30
G6.C	23,382,954	24,569,078	47,952,032	51.23

Supplementary table 2: Benchmarking of Universal Single Copy Orthologs (BUSCO) statistics for *A. americanum* midgut at each feeding stage.

Sample	Complete (%)	Single (%)	Duplicate (%)	Fragmented (%)	Missing (%)
UF	60.6	54.9	5.7	4.9	34.5
G1	66.9	59.8	7.1	5.2	27.9
G2	64	57.5	6.5	4.9	31.1
G3	64.1	57.5	6.6	4.9	31
G4	52.1	46.4	5.7	4.1	43.8
G5	52.4	47.2	5.2	4.3	43.3
G6	59.3	53.6	5.7	4.8	35.9

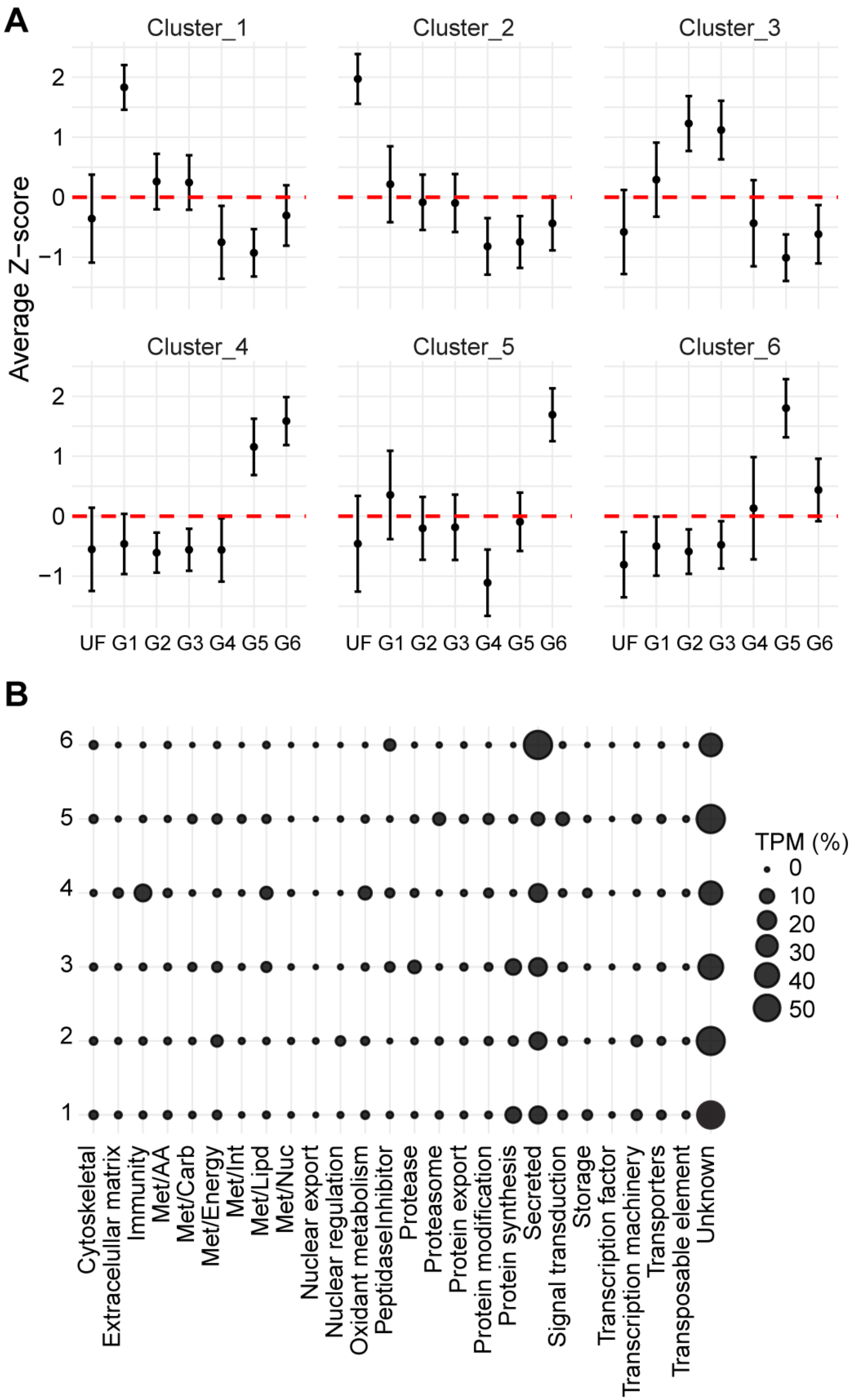
A
Cluster_1
Cluster_2
Cluster_3
Cluster_4
Cluster_5
Cluster_6
Average Z−score
2
1
0
−1
UF G1 G2 G3 G4 G5 G6
B
TPM (%)
0
10
20
30
40
50
6
5
4
3
2
1
Cytoskeletal
Extracelullar matrix
Immunity
Met/AA
Met/Carb
Met/Energy
Met/Int
Met/Lipd
Met/Nuc
Nuclear export
Nuclear regulation
Oxidant metabolism
PeptidaseInhibitor
Protease
Proteasome
Protein export
Protein modification
Protein synthesis
Secreted
Signal transduction
Storage
Transcription factor
Transcription machinery
Transporters
Transposable element
Unknown

Supplementary figure 1: Unsupervised clustering of transcripts identified in *A. americanum* midgut at different feeding stages. **(A)** The dots represent the average Z-score of the transcript per million (TPM) from the transcripts within each cluster and the error bars represent the standard deviation of the mean. The red dotted line marks the zero position in the y-axis. **(B)** Bubble plot representing the abundance of the transcript functional class identified in each cluster.

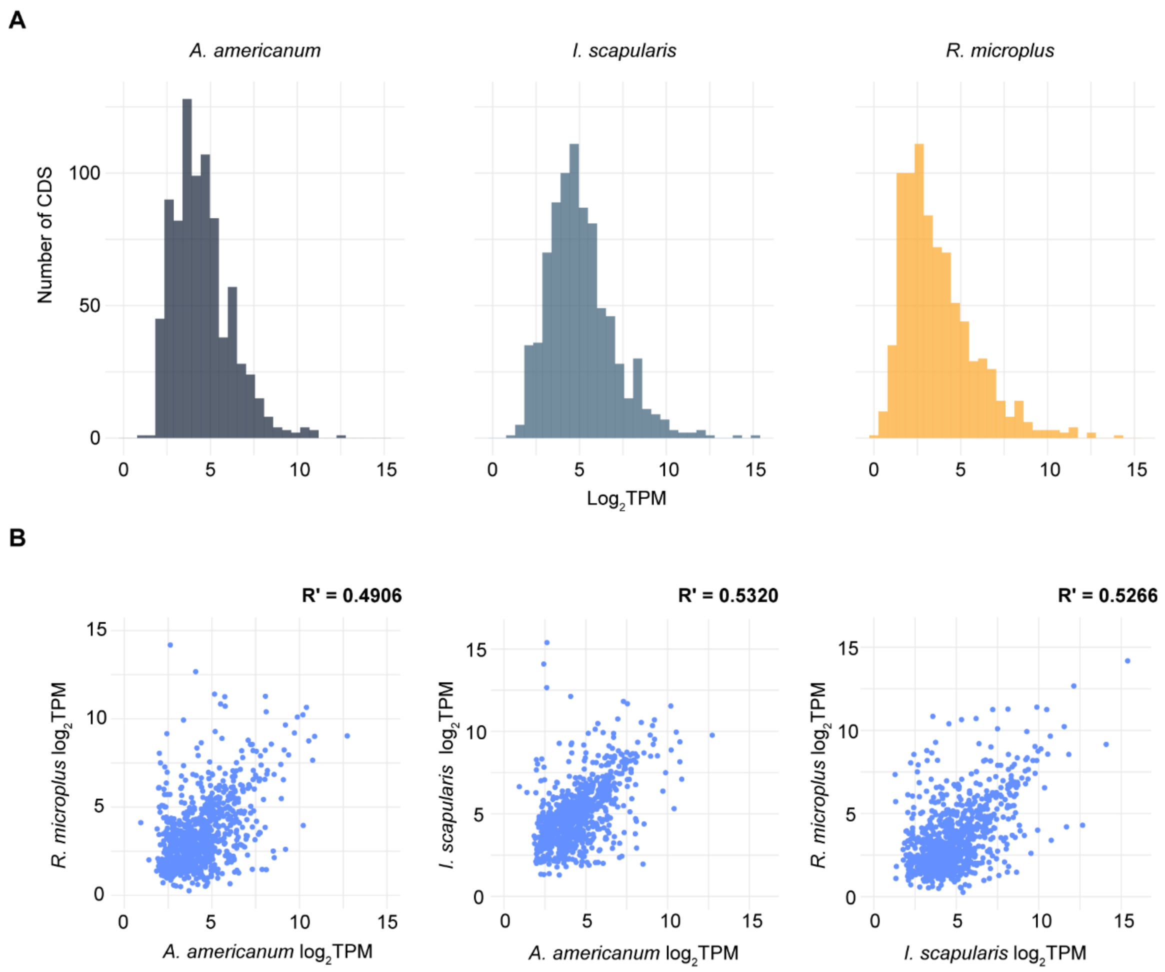

Supplementary figure 2: Comparison of the shared transcripts within the midgut of *A. americanum*, *I. scapularis*, and *R. microplus* during the slow-feeding stage. **(A)** Histograms of the 823 shared transcripts among the three tick species based on their Log_2TPM values. **(B)** Scatter plot of the Log_2TPM of the 823 shared transcripts in the midgut of slow-feeding ticks. The Pearson correlation coefficient (R^2) was estimated of each pair-wise comparison.

A

```
                  *        20         *        40         *
Amseq_4908 : MAATQPRQNYHIDCEARINKQINMELYASYVYTSMAYYFDRDDVALPGFH :  50
Dsilvarum  : MAATQPRQNYHVDCEARINKQINMELYASYVYTSMAYYFDRDDVALPGFH :  50
Rsanguineu : MAATQPRQNYHVDCEARINKQINMELYASYVYTSMAYYFDRDDVALPGFH :  50
Acajennens : MAATQPRQNYHVDCEARINKQINMELYASYVYTSMAYYFDRDDVALPGFH :  50
Aparvum    : MAATQPRQNYHIDCEARINKQINMELYASYVYTSMAYYFDRDDVALPGFH :  50
Aaureolatu : MAATQPRQNYHIDCEARINKQINMELYASYVYTSMAYYFARDDVALPGFH :  50

                      60         *        80         *       100
Amseq_4908 : KFFKKCSEEEREHADKLMKYQKMRGGRVVLQPIQKPSQDEWGTGLEAMQA : 100
Dsilvarum  : KFFKKSSEEEREHAEKLMKYQNMRGGRVVLQPIQKPAQDEWGAGLDAMQA : 100
Rsanguineu : KFFKKSSEEEREHAHKLMKYQNMRGGRVVLQPIQKPSQDEWGAGLDAMQA : 100
Acajennens : KFFKKSSEEEREHADKLMKYQNMRGGRVVLQPIQKPSQDEWGTGLEAMQA : 100
Aparvum    : KFFKKSSEEEREHADKFMKYQNMRGGRVVLQPIQKPSQDEWGTGLEAMQA : 100
Aaureolatu : KFFKKSSEEEREHADKLMKYQNMRGGRVALQPIQKPSQDEWGTGLEAMQA : 100

                  *       120         *       140         *
Amseq_4908 : ALELEKAVNQSLLDLHKLATDHNDAQ------------------------ : 126
Dsilvarum  : ALELEKTVNQSLLDLHKLATDHNDAQLCDFLESEYLAEQVKSIKELSDYV : 150
Rsanguineu : ALELEKTVNQSLLDLHKLATDHNDAQLCDFLESEYLEEQVKAIKELSDYV : 150
Acajennens : ALELEKTVNQSLLDLHKLATDHNDAQLCDFLESEYLAEQVKAIKELSDYV : 150
Aparvum    : ALELEKTVNQSLLDLHKLATDHNDAQLCDFLESEYLAEQVKAIKELSDYV : 150
Aaureolatu : ALELEKTVNQSLLDLHKLATDHNDAQLCDFLESEYLAEQVKAIKELSDYV : 150

                    160         *
Amseq_4908 : ---------------------- :   -
Dsilvarum  : TNLKRVGPGLGEYMFDKETLSD : 172
Rsanguineu : TNLKRVGPGLGEYMFDKETLSD : 172
Acajennens : TNLKRVGPGLGEYMFDKETLSD : 172
Aparvum    : TNLKRVGPGLGEYMFDKETLSD : 172
Aaureolatu : TNLKRVGPGLGEYMFDKETLGD : 172
```

B

>Amseq_49087 putative ferritin, type-1

GGAATAAGCAGCTCCTGGAACAGCGGCCGGACGGTATAAAA
GCGCGCTCCCCGCCATTTCCGCCATGTTTTGGTCGATG**TTA**
TGCTTCAACAGTGATTGAACGAGCATCGCCAAACTGTTTCA
CTCTCTCCGAACTCTTCCCAAATCCTTCTTGAATACAAGTA
CAGTTTGAGATTCCGGACGGGAACAAGACGACGAAG**ATG**GC
CGCCACTCAGCCCCGCCAAAACTACCACATCGATTGCGAGG
CCCGCATCAACAAGCAGATCAACATGGAACTCTACGCGAGC
TACGTCTACACTTCAATGGCCTACTACTTCGACCGTGACGA
TGTGGCCCTGCCGGGATTTCACAAGTTCTTCAAGAAGTGTA
GTGAGGAGGAGCGTGAGCATGCCGATAAGCTGATGAAGTAC
CAGAAAATGCGTGGAGGGCGTGTTGTGCTGCAGCCAATCCA
GAAGCCGTCGCAGGACGAATGGGGCACTGGCCTGGAGGCTA
TGCAGGCCGCTCTGGAGCTGGAGAAGGCTGTCAACCAGTCG
CTGCTTGACCTGCACAAGCTGGCTACTGACCACAACGATGC
TCAG

C

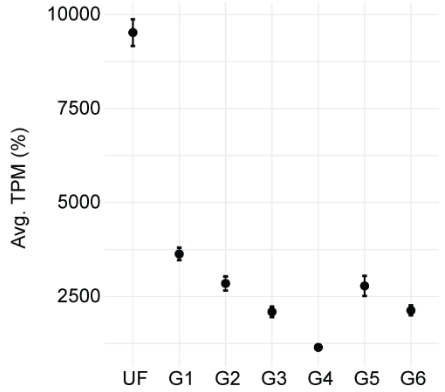

Supplementary figure 3: Primary sequence analysis of the putative type 1 ferritin

(Amseq_49087) identified in the midgut of unfed adult females *A. americanum* ticks. **(A)** Amino acid alignment of Amseq_49087 with other putative type 1 ferritins from *A. cajennenses* (JAC20482.1), *A. parvum* (JAC25210.1), *A. aureolatum* (JAT93262.1), *R. sanguineus* (AAQ54715.1) and *D. silvarum* (XP_037568710.1). **(B)** Nucleotide sequence of Amseq_49087. The iron-responsive element (IRE) is highlighted in blue and the start codon in red. **(C)** Transcriptional kinetic of Amseq_49087 in the midgut of adult females *A. americanum* ticks at different feeding stages. The dots represent the average transcript per million (TPM) in each biological condition and the error bars represent the standard deviation of the mean.

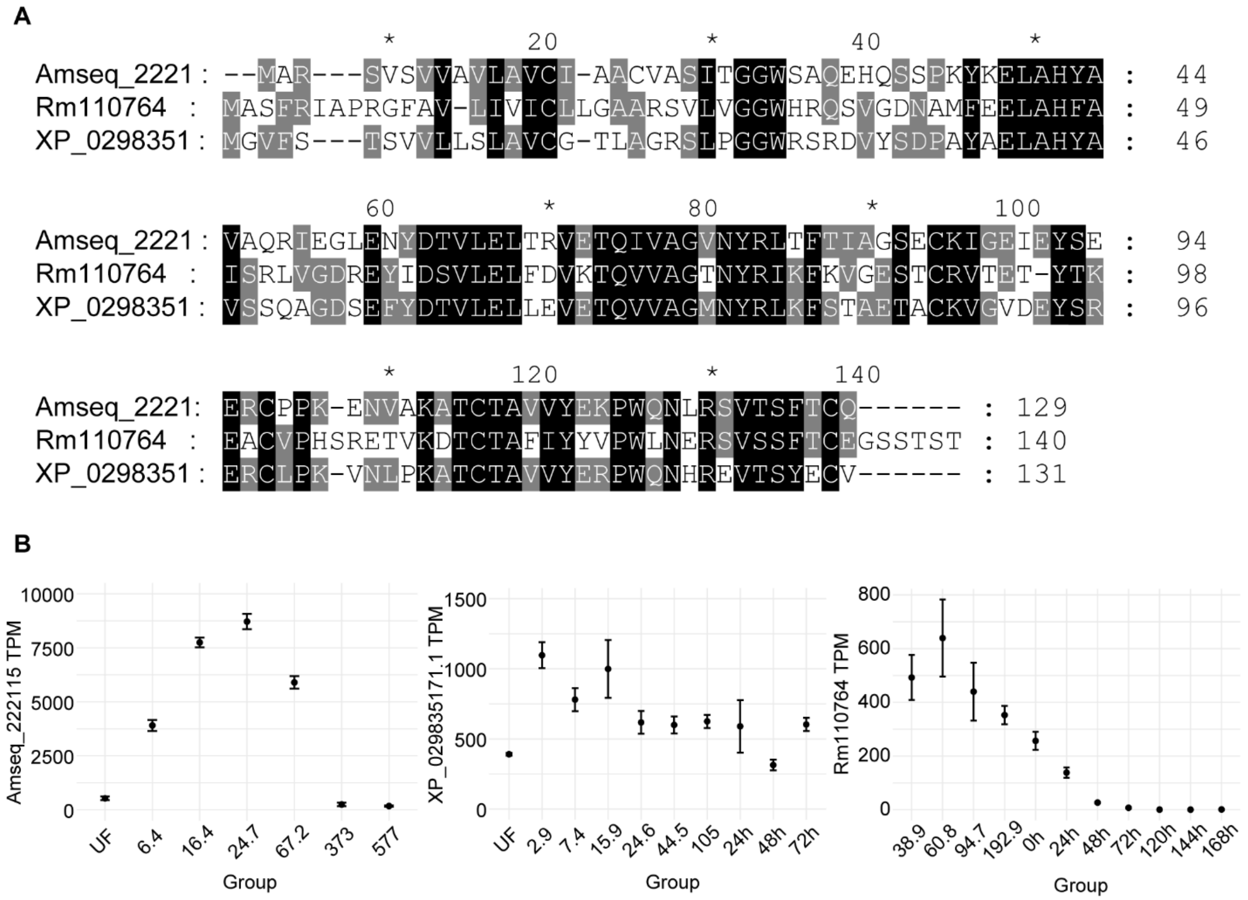

Supplementary figure 4: Comparison of putative type-2 cystatins abundant during the slow-feeding phase of *A. americanum*, *I. scapularis* and *R. microplus* adult females. **(A)** amino acid alignment of Amseq_222115 (*A. americanum*), XP_029835171.1 (*I. scapularis*) and Rm110764 (*R. microplus*). Identical (black) and similar (gray) residues are highlighted. (**B)** Transcriptional profile of the three cystatins during the different feeding stages of adult female ticks. Data from XP_029835171.1 and Rm110764 were obtained from [20,21]. Groups represent unfed (UF), partially fed ticks grouped by their average weight (mg) or hours post-detachment (0h – 168h).

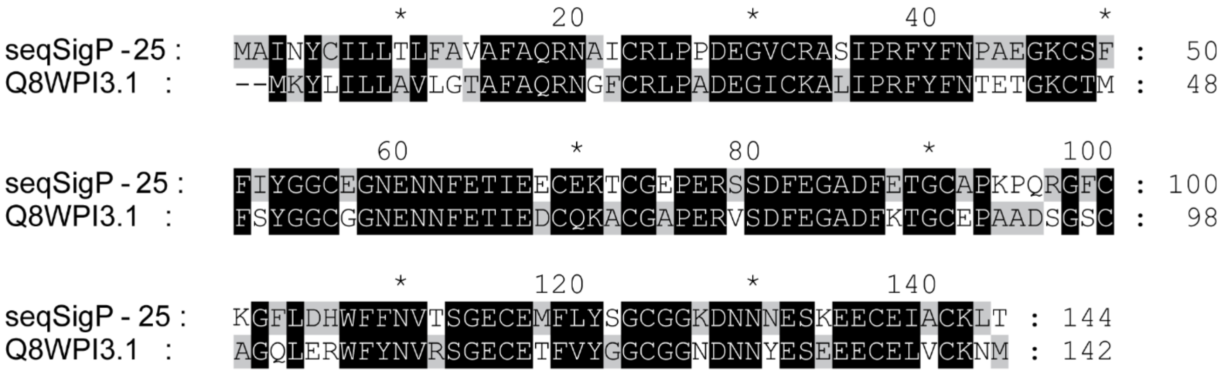

Supplementary figure 5: Amino acid alignment of seqSigP-25462 from *A. americanum* and boophilin (Q8WPI3.1) from *R. microplus*. Identical (black) and similar (gray) residues are highlighted.

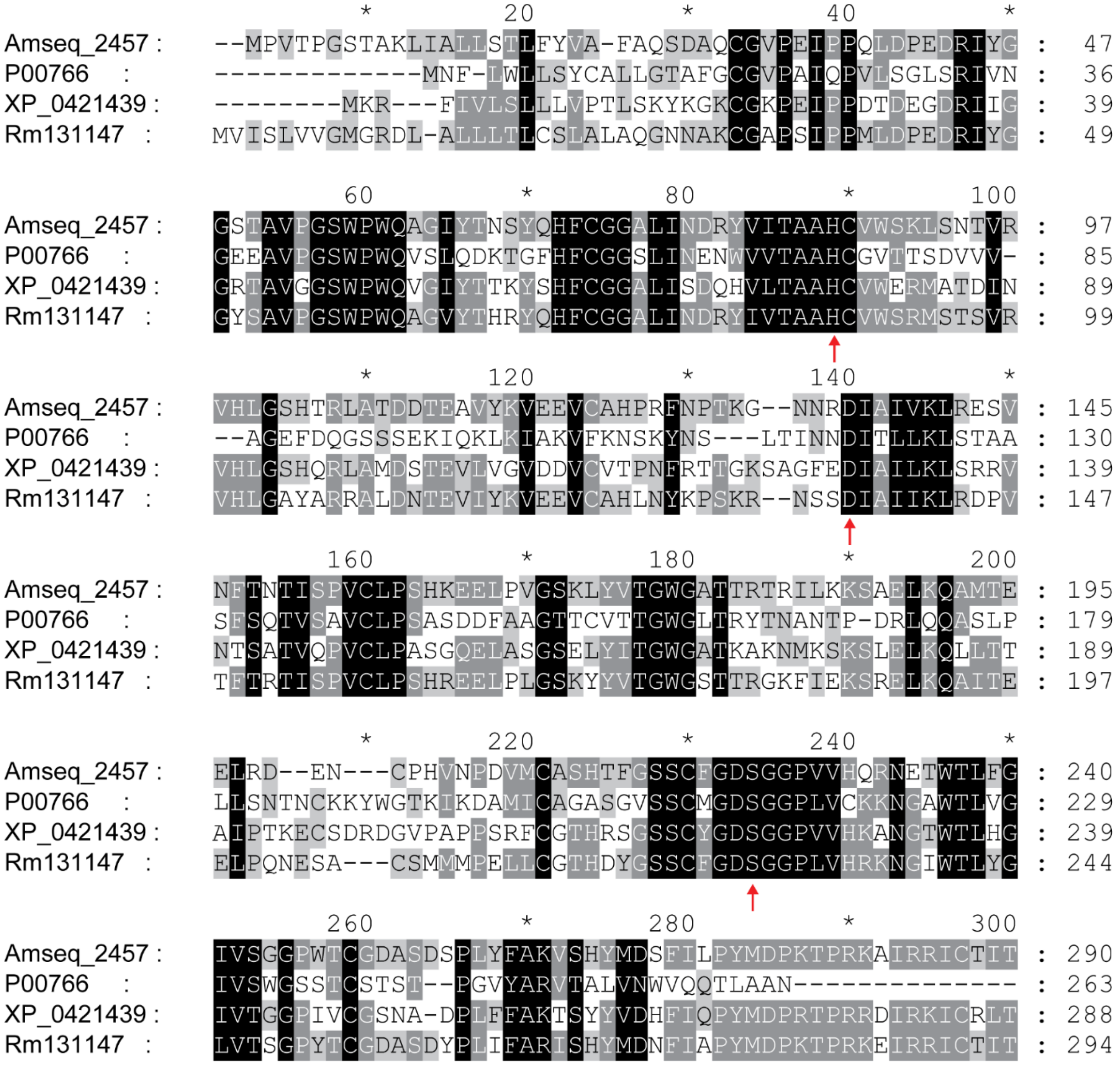

Supplementary figure 6: Amino acid alignment of serine peptidases from *Bos taurus* (P00766), *I. scapularis* (XP_042143948.1), *A. americanum* (Amseq_245718) and *R. microplus* (Rm131147). Identical (black) and similar (gray) residues are highlighted, while the putative catalytic residues are indicated by the red arrows.

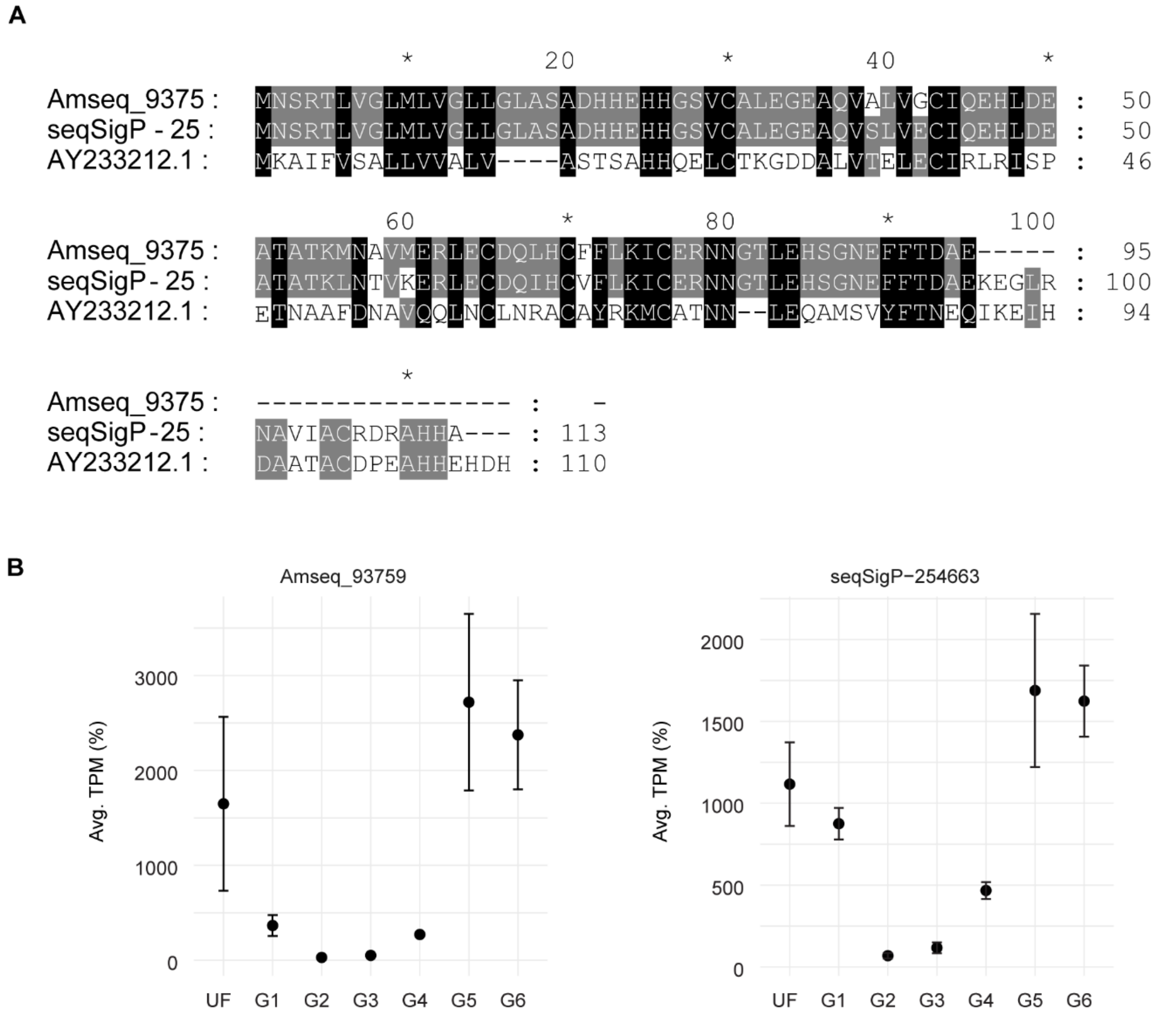

Supplementary figure 7: (A) Amino acid alignment of *R. microplus* microplusin (GenBank: AY233212.1) and the predicted amino acid sequence of *A. americanum* microplusin-like transcripts (Amseq_93759 and seqSigP-254663). Identical (black) and similar (gray) residues are highlighted. **(B)** Transcriptional profile of Amseq_93759 and seqSigP-254663. Dots represent the average TPM at each feeding stage, while error bars represent the standard deviation of the mean.

Supplementary file 1: Windows-compatible hyperlinked Excel file containing the functional annotation of the 15,999 putative CDS identified in *A. americanum* midgut at different feeding stages.

Supplementary file 2: Excel file containing the accession numbers of the shared transcripts within the midgut of unfed and slow-feeding *I. scapularis*, *R. micrplus* and *A. americanum* adult females.
